# Immunomagnetic B cell isolation as a tool to study blood cell subsets and enrich B cell transcripts

**DOI:** 10.1186/s13104-021-05833-z

**Published:** 2021-11-18

**Authors:** Amanda N. Henning, Daniel Green, Ryan Baumann, Patrick Grandinetti, Steven L. Highfill, Huizhi Zhou, Valeria De Giorgi

**Affiliations:** 1 Department of Transfusion Medicine, Clinical Center, National Institutes of Health, Bethesda, MD USA; 2 Women’s Malignancies Branch, National Cancer Institute, National Institutes of Health, Bethesda, MD USA; 3 Experimental Transplantation and Immunology Branch, National Cancer Institute, National Institutes of Health, Bethesda, MD USA

**Keywords:** RNA-sequencing, B cells, Differential gene expression, Gene signature

## Abstract

**Objective:**

Transcriptional profiling of immune cells is an indispensable tool in biomedical research; however, heterogenous sample types routinely used in transcriptomic studies may mask important cell type-specific transcriptional differences. Techniques to isolate desired cell types are used to overcome this limitation. We sought to evaluate the use of immunomagnetic B cell isolation on RNA quality and transcriptional output. Additionally, we aimed to develop a B cell gene signature representative of a freshly isolated B cell population to be used as a tool to verify isolation efficacy and to provide a transcriptional standard for evaluating maintenance or deviation from traditional B cell identity.

**Results:**

We found RNA quality and RNA-sequencing output to be comparable between donor-matched PBMC, whole blood, and B cells following negative selection by immunomagnetic B cell isolation. Transcriptional analysis enabled the development of an 85 gene B cell signature. This signature effectively clustered isolated B cells from heterogeneous sample types in our study and naïve and memory B cells when applied to transcriptional data from a published source. Additionally, by identifying B cell signature genes whose functional role in B cells is currently unknown, our gene signature has uncovered areas for future investigation.

**Supplementary Information:**

The online version contains supplementary material available at 10.1186/s13104-021-05833-z.

## Introduction

The rise of increasingly affordable and accessible next-generation sequencing (NGS) technologies has made transcriptional profiling via RNA-sequencing (RNA-seq) an achievable and essential research tool [1]. Whole blood (WB) and peripheral blood mononuclear cells (PBMC) are routinely used sample types in RNA-seq studies due to their ease of collection and capacity for long-term storage. However, while they provide a wealth of biological information, the heterogeneity of these sample types can be a drawback, as cell type-specific transcriptional differences can be masked in bulk RNA-seq approaches [2].

When transcriptional information on a discrete cell type is required, multiple experimental techniques are available to overcome sample heterogeneity, including single cell sequencing and fluorescence-activated cell sorting (FACS). While these techniques are highly effective, they require specialized equipment and can be costly [3]. The most feasible approach for many labs is the use of immunomagnetic separation methods, as it is cost-effective, requires minimal specialized training or equipment, and many commercially available kits exist for isolating standard immune cell components [4].

B cells are an immune cell type of particular interest in biomedical research, representing a cornerstone of adaptive immunity, with direct involvement in certain cancers and autoimmune disorders [5, 6]. There have been a number of studies identifying B cell gene expression patterns associated with various disease states, including non-Hodgkin’s lymphoma [7] and autoimmune diseases [8]. Fewer studies have attempted to identify a B cell gene signature from healthy donors relative to a heterogenous population [9, 10], and most of these studies were performed using microarrays, which can be limited in their sensitivity and reproducibility [11]. With the increasing importance of subset-specific transcriptional analysis in disease research, having a high-quality B cell gene signature obtained via modern NGS technologies is critical. In this study, we have evaluated the RNA quality and sequencing output of B cells isolated using negative selection immunomagnetic cell separation and have established a baseline B cell gene signature from healthy donors. Our B cell signature provides a useful tool for verifying B cell purity at the transcriptional level and establishes a transcriptional baseline for assessing deviations incurred by environmental or experimental perturbations. Furthermore, our signature has identified genes that play an unknown role in B cell function that are of interest for future investigations.

## Main text

### Methods

#### Sample collection, processing, and NGS

Human whole blood samples were obtained from healthy donors on an IRB-approved NIH protocol (99-CC-0168). PBMC were isolated from whole blood using Ficoll-Paque Plus solution (GE Healthcare), and B cells were subsequently isolated using the EasySep™ Human B cell Isolation kit (StemCell Technologies). RNA was isolated from 200 µL whole blood using the Quick-RNA Whole Blood kit (Zymo Research), and RNA was isolated from PBMC and B cells using the RNeasy Plus Mini kit (Qiagen). RNA was assessed for quality on an Agilent 2100 Bioanalyzer (Agilent Technologies). Library preparations were done using TruSeq Stranded mRNA Library Prep (Illumina). RNA input was 300 ng for WB and PBMC samples and 100 ng for isolated B cells. Libraries were normalized to 10 nM, and equal volumes of all 12 libraries were pooled together for sequencing on a NextSeq 550 instrument (Illumina).

#### Bioinformatic and statistical analysis

Sequenced reads were aligned to the human reference genome (UCSC hg19) using the RNA-Seq Alignment application (v2.0.1) on the BaseSpace Sequencing Hub (Illumina). Sequencing files are available on the GEO repository: GSE186768. Differential expression analysis was performed in R (v4.0.2) [12] using the DESeq2 package (v1.28.1). Genes were considered to be differentially expressed if they had a log2 fold change of < − 1 or > 1 and an adjusted p-value (padj) of < 0.05. Bioinformatic analyses were performed using the topGO R package (v2.40.0), the web-based WebGestalt analysis tool (www.webgestalt.org) [13], and the GSEA (v4.0.3) desktop application [14, 15]. For validation of our B cell gene signature, the RNA-seq dataset from Monaco et al. [16] was downloaded from GEO: GSE107011. The ImmGen database (https://www.immgen.org/) was used to investigate signature gene expression levels. Additional statistics and figure creation were done using GraphPad Prism (v8.4.3; GraphPad Software). For a more detailed methodological description, see Additional file 1.

### Results and discussion

#### RNA quality and NGS output is consistent among WB, PBMC and isolated B cells

**Fig. 1:**
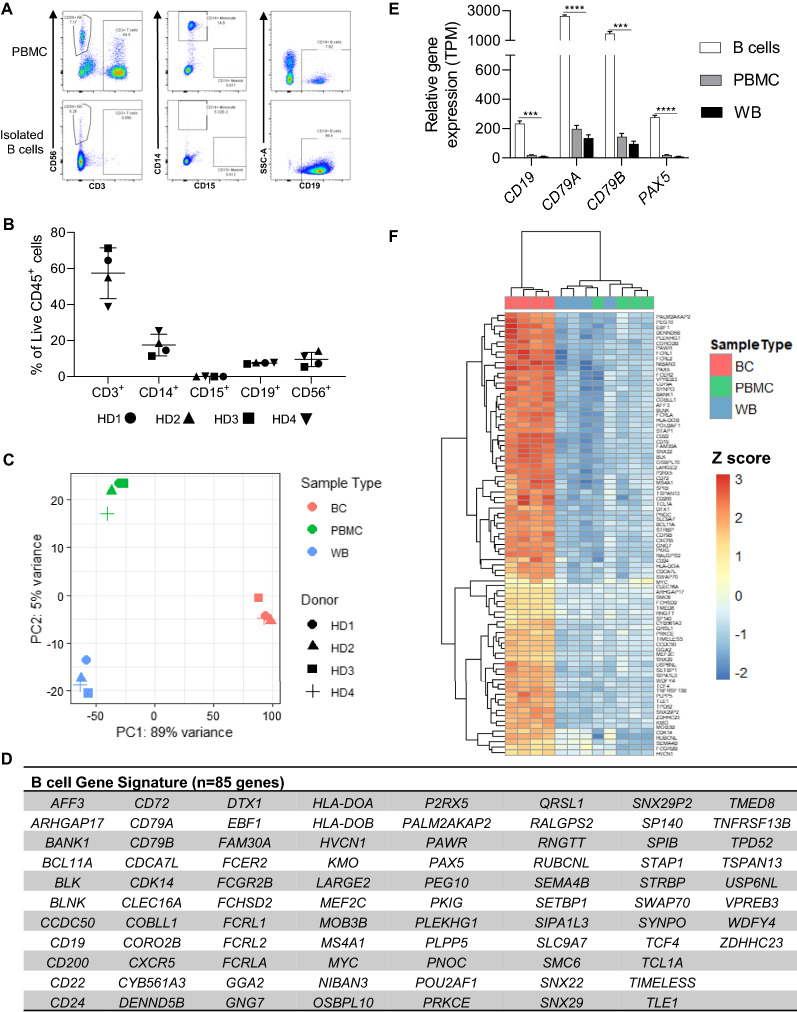
Identification of a B cell gene signature. **a** Representative flow plots of PBMC immunophenotyping (top) and B cell purity check (bottom). Cells were previously gated on Lymphocytes/Single cells/Live/CD45+. **b** Summary of immunophenotyping results for PBMC samples. Individual values are displayed along with the average ± SD. **c** PCA plot from transcriptional analysis of whole blood (WB), PBMC and isolated B cells (BC). **d** List of 85 genes that make up the B cell gene signature. **e** Relative gene expression of select B cell signature genes. Statistics computed using unpaired T tests with correction for multiple comparisons. Graph displays average ± SD. p-value applies to comparisons of B cells versus both PBMC and WB. **f.** Heatmap with hierarchical clustering using the B cell gene signature. *p < 0.05; **p < 0.01; ***p < 0.001; ****p < 0.0001

To compare the quality of RNA and sequencing data generated from related sample types, we collected donor-matched WB and PBMC from four healthy donors (HD). Immunophenotyping of PBMC revealed some inter-donor variability (Fig. 1a, b; Additional file 2: Fig. S1a); however, the CD19^+^ B cell population was consistent at 7%. B cells were then isolated from fresh PBMC using the StemCell™ EasySep™ Human B cell isolation kit. This kit utilizes negative selection to isolate B cells. In this way, non-B cells are labeled with antibodies conjugated to magnetic particles, and the cells remaining after magnetic separation constitute an enriched B cell population. B cell purity was assessed via flow cytometry in all samples, and we observed a robust enrichment of B cells, with > 98% of CD45^+^ lymphocytes expressing CD19 (Fig. 1a; Additional file 2: Fig. S1b). RNA was isolated from WB, PBMC and B cells, and RNA quality was assessed based on RNA integrity number (RIN). All samples demonstrated high RIN scores (RIN > 8) suitable for NGS studies, and yielded sufficient RNA for NGS library preparation (Table 1).

**Table 1:** RNA quality and NGS output among sample types

RNA quality and yield	Whole blood	PBMC	Isolated B cells
RIN	9.28 (0.3)	9.75 (0.3)	9.78 (0.2)
RNA yield (ng)	301 (30.9)	559 (364.2)	186 (137.4)
Sequencing output			
Total reads (× 10^6^)	53.97 (7.9)	47.21 (2.45)	57.31 (4.86)
Abundant reads (% of total)	7.60 (1.33)	7.46 (1.70)	9.87 (1.19)
Aligned reads (% filtered)	97.60 (0.33)	97.88 (0.28)	94.93 (1.74)
Fold coverage (coding)	113.32 (20.53)	103.92 (6.54)	100.69 (11.92)

Values represent mean (SD), n = 4/group. RNA yield for whole blood corresponds to ng/200 µl, and for PBMC and B cells yield represents ng/1e6 cells. RIN, RNA integrity number

Samples were sequenced on the Illumina NextSeq550 platform. The total number of reads/samples, percent of reads mapping to abundant regions of the genome, percent aligned reads, and fold coverage across coding regions were relatively consistent across different sample types (Table 1). Overall, negative selection B cell isolation was highly efficient and resulted in RNA and sequencing output that was of similar quality among sample types. In this way, the additional processing steps required for B cell isolation did not adversely affect experimental results.

#### Identification of a B cell gene signature

RNA-sequencing was performed on freshly isolated, donor-matched WB, PBMC, and B cells (Additional file 3: Table S1). Principle component analysis (PCA) demonstrated tight and distinct clustering of sample types, irrespective of donor (Fig. 1c), and differential expression analysis identified 7027 differentially expressed genes (DEGs) between B cells and WB and 5,537 DEGs between B cells and PBMC (Additional file 3: Table S2). Significant gene ontology (GO) terms related to B cell-specific functions were identified, and gene set enrichment analysis (GSEA) showed positive enrichment of B cell-specific gene sets (Additional file 2: Fig. S2a, b; Additional file 3: Table S3). Bioinformatic analyses, therefore, supported efficient B cell isolation and transcriptional capture.

Differential gene expression analysis in PBMC and B cell samples was used to identify a robust B cell gene signature indicative of a homogenous population derived from healthy individuals. The top 200 most significant DEGs between B cells and PBMC effectively clustered all samples and included 51 upregulated genes and 149 downregulated genes (Additional file 2: Fig. S2c). To expand our signature gene set, these 51 upregulated genes were combined with a B cell transcriptional module [17] to create a B cell gene signature consisting of 85 genes upregulated in B cells relative to heterogenous PBMC samples (Fig. 1d). The module from Chaussabel et al. [17] was derived using computationally identified patterns of coordinately expressed genes in PBMC microarrays sourced from multiple disease states. We felt that combining gene sets identified using these two divergent methodological approaches would create a robust B cell gene signature. Indeed, this gene set was highly enriched for B cell-specific GO terms (Additional file 2: Fig. S3a), included a number of highly expressed genes with critical B cell functions (Fig. 1e; Additional file 2: Fig S3b), and resulted in distinct clustering of B cell samples relative to heterogenous sample types (Fig. 1f).

#### Validation of B cell gene signature in external dataset

**Fig. 2:**
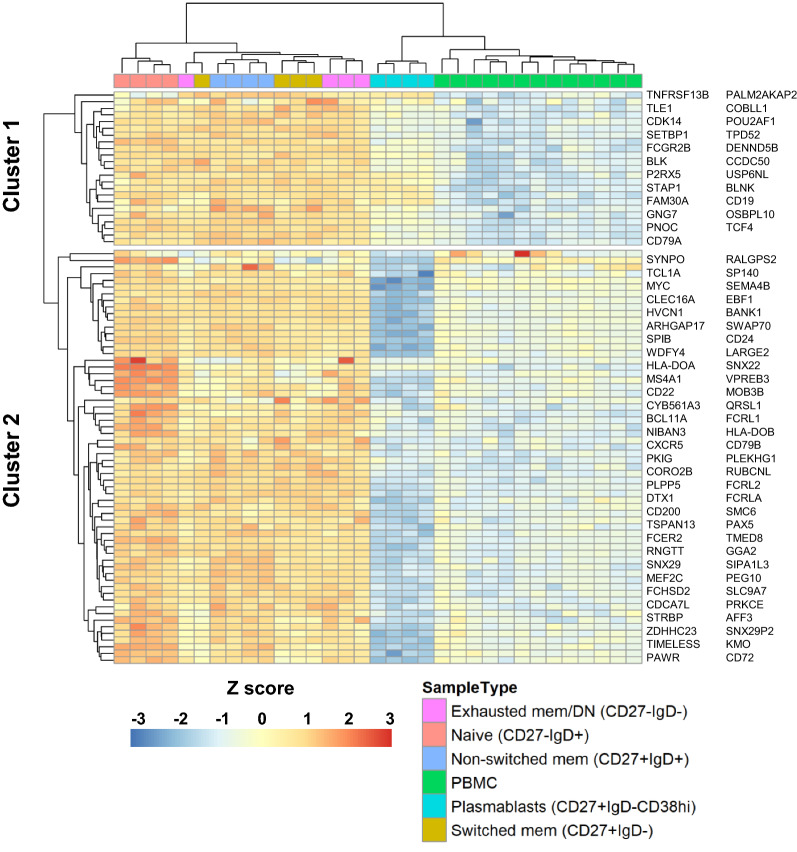
Validation of B cell gene signature. Heatmap with hierarchical clustering using the B cell gene signature and RNA-seq data from Monaco et al. (16). B cell subsets are indicated at the top, and cell surface markers used for sorting are listed in the legend. Genes corresponding to each cluster are listed on the side, and gene order corresponds to their order in the heatmap. DN, double negative

To validate our B cell gene signature, we used transcriptional data published by Monaco and colleagues [16] consisting of RNA-seq data from sorted immune cell types, including multiple B cell subsets. B cell subsets were isolated via FACS and were classified as: naïve (CD27^−^IgD^+^), non-switched memory (CD27^+^IgD^+^), switched memory (CD27^+^IgD^−^), exhausted memory or double negative (DN) (CD27^−^IgD^−^), and plasmablasts (CD27^+^IgD^−^CD38^hi^). As the immunomagnetic separation method we used did not discriminate between specific B cell subsets, we wanted to ensure that our B cell signature was indicative of these multiple B cell developmental states. Hierarchical clustering using our B cell gene signature was performed on log2-transformed Transcripts Per Million (TMP) data. Our B cell signature effectively clustered naïve and memory B cell populations from heterogenous PBMC samples (Fig. 2); however, plasmablasts demonstrated a divergent gene expression profile. This is to be expected since, compared to naïve and memory B cell, plasmablasts are a small component of the circulating B cell population (96% vs ~ 1%) [18], and thus contributed little to our B cell signature. Furthermore, plasmablasts, along with plasma cells, represent a highly specialized B cell subset with a unique transcriptional profile. They have been shown to downregulate a number of traditional B cell genes, including *CD24*, *CXCR5*, *PAX5*, *EBF1*, and *SPIB* [19], all of which were part of a downregulated cluster (Fig. 2) in our analysis. Since some genes crucial for B cell function can also be expressed in other immune cell types, we wanted to verify that our B cell gene signature would robustly identify B cells when challenged with other homogenous non-B cell populations. Hierarchical clustering was performed on the same external dataset [16], this time using sorted B cell subsets, heterogenous PBMC samples, and 24 additional sorted leukocyte subsets. Once again, our B cell signature effectively clustered naïve and memory B cell populations separate from all other leukocyte cell types (Additional file 2: Fig. S4). Overall, a validation of our B cell gene signature using an external dataset found it to be indicative of naïve and memory B cell subsets relative to both a heterogenous PBMC population as well as homogenous non-B cell leukocyte populations.

#### Manual characterization of B cell signature genes

An in-depth exploration of B cell signature genes revealed that over half have known functional roles in B cells (Additional file 2: Fig. S5). The remaining genes had no reported function in B cells; however, they represent promising areas of further investigation, as their significant expression in B cells suggests involvement in functional pathways. Indeed, *CCDC50*, *KMO*, *PAWR*, *PEG10*, and *PLPP5* may play a role in multiple B cell-associated cancers [20–24], and *CDCA7L* and *OSBPL10* may be risk markers in multiple myeloma and diffuse large B cell lymphoma, respectively [25, 26]. Additionally, investigation of B cell signature gene expression levels via the Immunological Genome Project (https://www.immgen.org/) Human Expression Data identified 39 genes as having B cell-specific gene expression (Additional file 2: Fig. S5, Additional file 3: Table S4). This included expected genes, such as many involved in the BCR signaling pathway, but also genes of unknown function in B cells, including *PLEKHG1*, *RALGPS2*, and *SYNPO*, among others. We have thus identified a B cell gene signature representative of a freshly isolated, homogenous B cell population consisting of both well-characterized B cell genes and novel genes whose functional characterization may provide insight in the understanding of B cell malignancies.

## Conclusions

RNA-sequencing performed in donor matched WB, PBMC, and isolated B cells has verified the use of negative selection immunomagnetic cell separation as a viable way to isolate B cells for NGS studies. We have identified a B cell gene signature representative of a freshly isolated, homogenous B cell population. In particular, our signature may be used for transcriptional verification of naïve or memory B cell identity, especially in instances where immunophenotyping is not possible, or to assess deviation from the traditional B cell transcriptome following chemical or genetic perturbation. Our B cell gene signature consists of many genes with well-characterized roles in B cell development and function; however, the identification of many genes with unknown B cell functions represents an important area for future investigations to enhance our understanding of B cell-related malignancies.

## Limitations

The limitations of this study include the relatively small sample size and our lack of B cell subset composition information for isolated samples. Additionally, we utilized a negative selection kit so as to avoid inadvertent B cell activation; however, it would be beneficial to experimentally verify the transcriptional effect of negative enrichment kits relative to positive enrichment kits or other forms of B cell isolation.

## Supplementary Information

**Additional file 1. **Extended methods.

**Additional file 2: Fig. S1.** Immunophenotyping of PBMC and isolated B cells. **Fig. S2.** Transcriptional analysis of donor-matched WB, PBMC, and isolated B cells. **Fig. S3.** Investigating the B cell gene signature. **Fig. S4.** Validation of B cell gene signature. **Fig S5.** Functional role of B cell signature genes.

**Additional file 3: Table S1.** TPM values across Sample Types. **Table S2.** Lists of differentially expressed genes between sample types. **Table S3.** Significantly enriched GO terms (p-value < 0.01) from upregulated DEGs in freshly isolated B cells vs PBMC and WB. **Table S4.** Investigating expression levels of B cell signature genes.

## Data Availability

The datasets generated and analyzed in the current study are available in the Gene Expression Omnibus (GEO) repository. RNA-seq data generated in this study can be found at GEO accession: GSE186768, and previously published data analyzed in this study can be found at GEO accession: GSE107011.

## Abbreviations

DEGs: Differentially expressed genes
DN: Double negative
FACS: Fluorescence-activated cell sorting
GO: Gene ontology
GSEA: Gene set enrichment analysis
HD: Healthy donor
NGS: Next-generation sequencing
PBMC: Peripheral blood mononuclear cells
RIN: RNA integrity number
RNA-seq: RNA sequencing
TPM: Transcripts per million
WB: Whole blood
