# Supplementary material for: Immunomagnetic B cell isolation as a tool to study blood cell subsets and enrich B cell transcripts
Source: BMC Res Notes. 2021 Nov 18;14:418. doi: 10.1186/s13104-021-05833-z (PMC8600718; doi:10.1186/s13104-021-05833-z)
Supplement: Supplementary file 1 — Additional file 1. Extended methods. [file 13104_2021_5833_MOESM1_ESM.docx]

**Additional File 1 – Extended methods**

**Donor samples**

Human whole blood samples were obtained from healthy donors on an IRB-approved NIH protocol (99-CC-0168). Research blood donors provided written informed consent (Clinical Trial number NCT00001846), and blood samples were de-identified prior to distribution. Demographically, healthy donor volunteers consisted of a 34yo male (HD1), 65yo male (HD2), 38yo male (HD3), and 58yo female (HD4).

**PBMC and B cell isolation**

PBMC were isolated from whole blood via density centrifugation using Ficoll-Paque Plus solution (GE Healthcare). Briefly, whole blood was overlaid onto Ficoll-Paque Plus and centrifuged at 1350rpm for 30 minutes at room temperature with the brake off. The interface layer containing PBMC was collected and washed twice in PBS, first at 400 x g and then at 200 x g, both for 10 minutes at room temperature. The resulting PBMC were counted on a Nexcelom Biosciences cell counter (Nexcelom Bioscience) using AOPI staining solution (Nexcelom Bioscience) to discriminate dead cells. B cells were isolated from PBMC using the EasySep Human B cell Isolation kit (StemCell Technologies) according to the manufacturer’s instructions.

**Immunophenotyping of PBMC and B cells**

Immunophenotyping of PBMC samples and the purity check of isolated B cells was done via flow cytometry. Cells were stained with: CD19 PE (BD; clone HIB19), CD14 FITC (BD; clone M5E2), CD15 BV510 (BD; clone W6D3), CD3 BV421 (BD; clone SK7), CD56 PE-Cy7 (BD; clone B157), and CD45 APC-Cy7 (BD; clone 2D1), with live/dead discrimination by 7-AAD staining (BioLegend). Samples were acquired on a CytoFlex flow cytometer (Beckman Coulter), and data were analyzed using FlowJo v10 (BD).

**RNA isolation**

RNA was isolated from 200µL whole blood using the Quick-RNA Whole Blood kit (Zymo Research) according to the manufacturer’s protocol. RNA was isolated from PBMC and B cells using the RNeasy Plus Mini kit (Qiagen) according to the manufacturer’s protocol. RNA was quantified on a NanoDrop 2000 (Thermo Scientific) and assessed for quality via the RNA integrity number (RIN) on an Agilent 2100 Bioanalyzer (Agilent Technologies).

**Library preparation and next-generation sequencing**

Library preparations were done using TruSeq Stranded mRNA Library Prep (Illumina) and barcoding was performed using TruSeq RNA Single Indexes Set A (Illumina) following manufacturer’s protocol. For WB and PBMC samples, an input of 300ng RNA was used for library preparation, and for isolated B cells 100ng of RNA was used. Library quality and size was assessed using an Agilent 2100 Bioanalyzer (Agilent Technologies), and KAPA Library Quantification Kits (Roche) were used for qPCR-based library quantification. Libraries were normalized to 10nM, and equal volumes of all 12 libraries were pooled together to be run on a single NextSeq 500/550 High Output flow cell (v2.5; Illumina). Sequencing was performed on a NextSeq 550 instrument (Illumina).

**Bioinformatic and statistical analysis**

Sequenced reads were aligned to the human reference genome (UCSC hg19) using the RNA-Seq Alignment application (v2.0.1) on the BaseSpace Sequencing Hub (Illumina), a cloud-based data analysis platform. This application utilized the STAR aligner, and transcript quantification was performed using Salmon. These analyses were conducted using the default parameters. Sequencing files are available on the Gene Expression Omnibus repository (GEO, <https://www.ncbi.nlm.nih.gov/geo/>): GSE186768. Differential expression analysis was performed in R (v4.0.2) (1) using the DESeq2 package (v1.28.1). The DESeqDataSet was designed to test for the effect of sample type while controlling for donor effects (design = ~Donor + SampleType). Genes were considered to be differentially expressed if they had a log2 fold change of <-1 or >1 and a Benjamini-Hochberg adjusted p-value (padj) of <0.05. Heatmaps, PCA plots and additional figures were created using the ggplot2 (v3.3.2), eulerr (v6.1.0), and pheatmap (v1.0.12) R packages. Gene Ontology analysis was done using the topGO (v2.40.0) R package and the web-based WebGestalt analysis tool ([www.webgestalt.org](http://www.webgestalt.org)) (2). Gene set enrichment analysis was performed using the GSEA (v4.0.3) desktop application (3, 4). For validation of our B cell gene signature, the RNA-seq dataset from Monaco *et al* (5) was downloaded from GEO: GSE107011. This included TPM data of heterogenous PBMC samples plus 29 sorted immune cell subsets categorized into 9 different sample types (B cells, CD4 T cells, CD8 T cells, dendritic cells, granulocytes, monocytes, NK cells, progenitor cells, and innate T cells). TPM data was log2(TPM + 1) transformed prior to heatmap generation. To investigate the B cell specificity of our B cell signature genes, we used human RNA-seq expression data from the Immunological Genome Project (ImmGen, <https://www.immgen.org/>). From this database, we collected normalized expression values from 20 different immune cell subsets, including three different B cell subsets, for all 85 B cell signature genes. A gene was considered to be B cell-specific if it demonstrated >10-fold increased expression over the median in only B cell subsets, or if the fold change over the median in B cell subsets was more than 5X that of the next highest cell type. Additional statistics were performed and figures were created using GraphPad Prism (v8.4.3; GraphPad Software). Specific statistical tests performed are described in the appropriate figure legends.

**Methods References:**

1. Team RC. R: A Language and Environment for Statistical Computing. 4.0.2 ed. Vienna, Austria: R Foundation for Statistical Computing; 2020.

2. Wang J, Duncan D, Shi Z, Zhang B. WEB-based GEne SeT AnaLysis Toolkit (WebGestalt): update 2013. Nucleic Acids Res. 2013;41(Web Server issue):W77-83.

3. Mootha VK, Lindgren CM, Eriksson KF, Subramanian A, Sihag S, Lehar J, et al. PGC-1alpha-responsive genes involved in oxidative phosphorylation are coordinately downregulated in human diabetes. Nat Genet. 2003;34(3):267-73.

4. Subramanian A, Tamayo P, Mootha VK, Mukherjee S, Ebert BL, Gillette MA, et al. Gene set enrichment analysis: a knowledge-based approach for interpreting genome-wide expression profiles. Proc Natl Acad Sci U S A. 2005;102(43):15545-50.

5. Monaco G, Lee B, Xu W, Mustafah S, Hwang YY, Carre C, et al. RNA-Seq Signatures Normalized by mRNA Abundance Allow Absolute Deconvolution of Human Immune Cell Types. Cell Rep. 2019;26(6):1627-40 e7.
