# Supplementary material for: Immunomagnetic B cell isolation as a tool to study blood cell subsets and enrich B cell transcripts
Source: BMC Res Notes. 2021 Nov 18;14:418. doi: 10.1186/s13104-021-05833-z (PMC8600718; doi:10.1186/s13104-021-05833-z)
Supplement: Supplementary file 2 — Additional file 2: Fig. S1. Immunophenotyping of PBMC and isolated B cells. Fig. S2. Transcriptional analysis of donor-matched WB, PBMC, and isolated B cells. Fig. S3. Investigating the B cell gene signature. Fig. S4. Validation of B cell gene signature. Fig S5. Functional role of B cell signature genes. [file 13104_2021_5833_MOESM2_ESM.pdf]

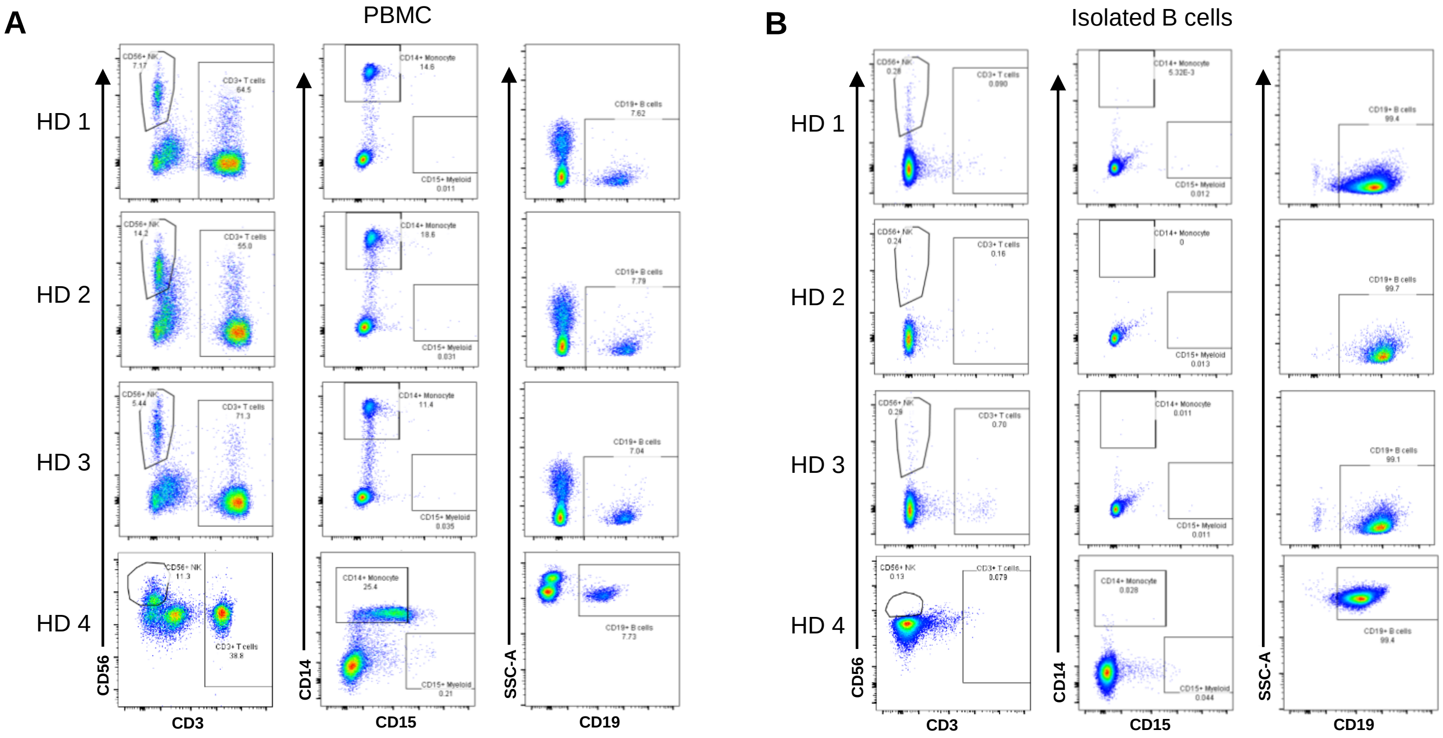

Figure S1: Immunophenotyping of PBMC and isolated B cells. **a.** Immunophenotyping of PBMC from Heathy Donors (HD) 1-4. **b.** Immunophenotyping of isolated B cells from HD1-4. In a and c, cells were previously gated on Lymphocytes/Single cells/Live/CD45+.

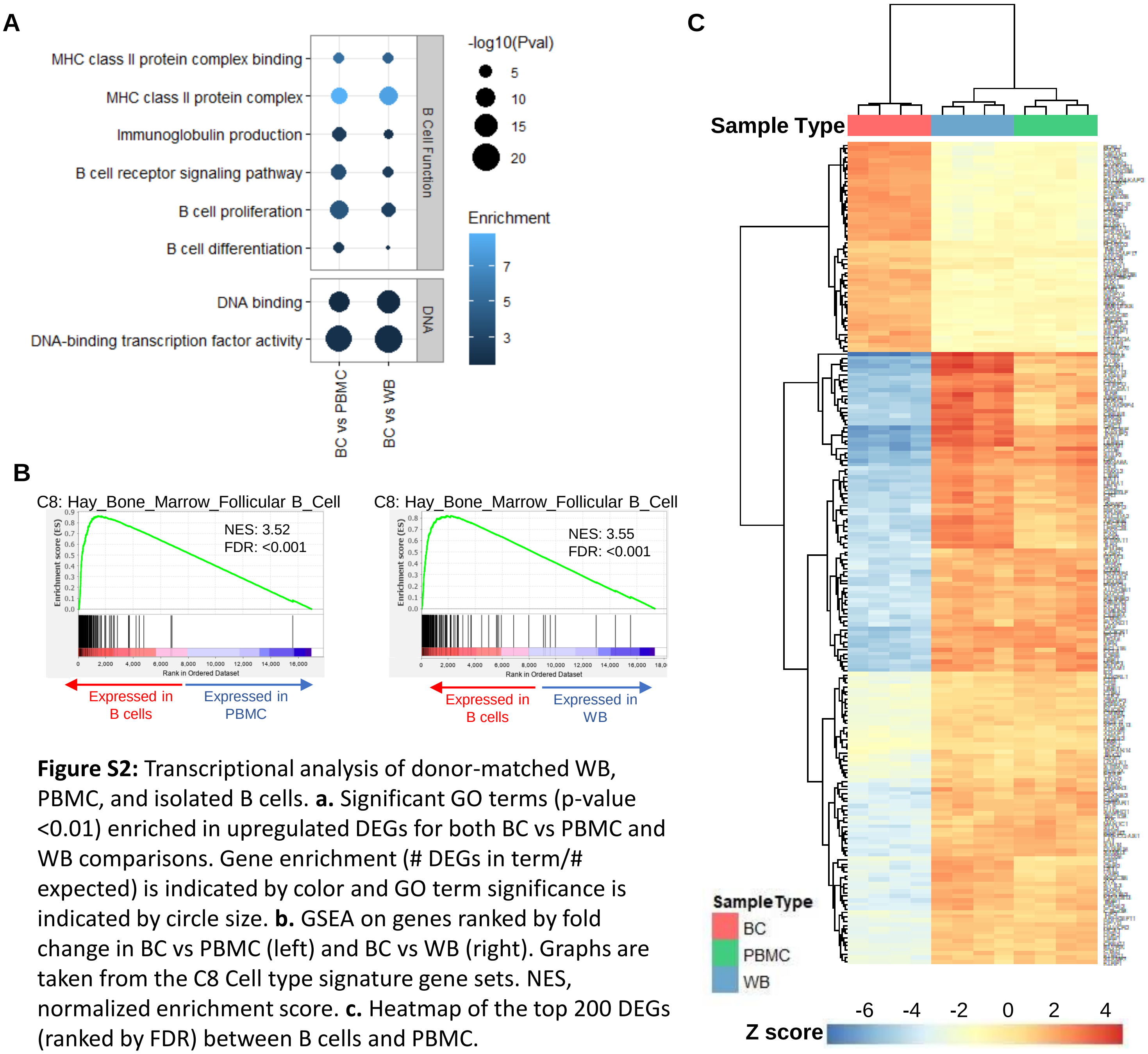

Figure S2: Transcriptional analysis of donor-matched WB, PBMC, and isolated B cells. **a.** Significant GO terms (p-value <0.01) enriched in upregulated DEGs for both BC vs PBMC and WB comparisons. Gene enrichment (# DEGs in term/# expected) is indicated by color and GO term significance is indicated by circle size. **b.** GSEA on genes ranked by fold change in BC vs PBMC (left) and BC vs WB (right). Graphs are taken from the C8 Cell type signature gene sets. NES, normalized enrichment score. **c.** Heatmap of the top 200 DEGs (ranked by FDR) between B cells and PBMC.

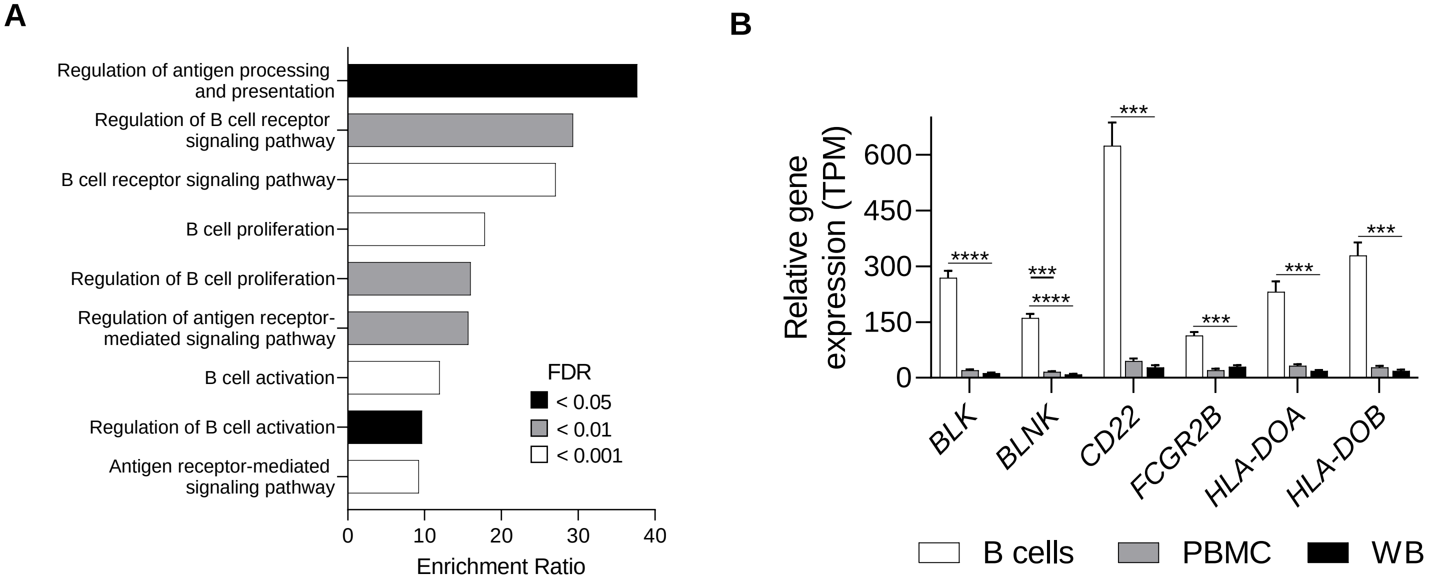

Figure S3: Investigating the B cell gene signature. **a.** GO analysis on the 85 genes that make up the B cell gene signature. Enrichment ratio (# DEGs in the term/# expected) is shown on the x-axis, and GO term significance is indicated by bar color (see legend). **b.** Relative gene expression of select genes from the B cell signature. Statistics computed using unpaired T tests with correction for multiple comparisons. Graph displays average ±SD. If only one p-value bar is shown, it applies to comparisons of B cells versus both PBMC and WB. *, p <0.05; **, p<0.01; ***, p<0.001; ****, p<0.0001.

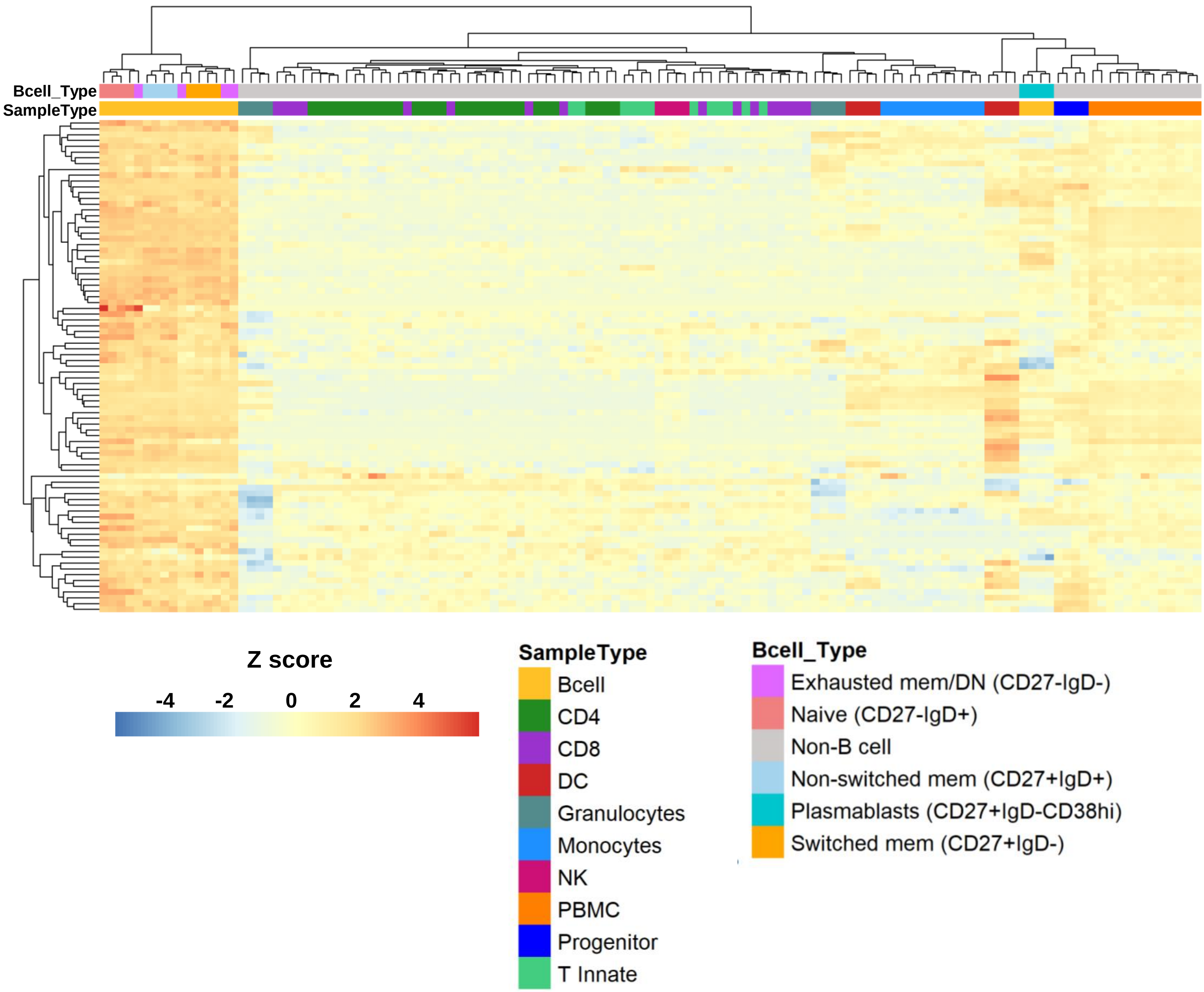

Figure S4: Validation of B cell gene signature. Heatmap with hierarchical clustering using the B cell gene signature and RNA-seq data from Monaco *et al* (ref 16). B cell subsets and leukocyte sample types are indicated at the top. Cell surface markers used for B cell sorting are listed in the legend. DN, double negative.

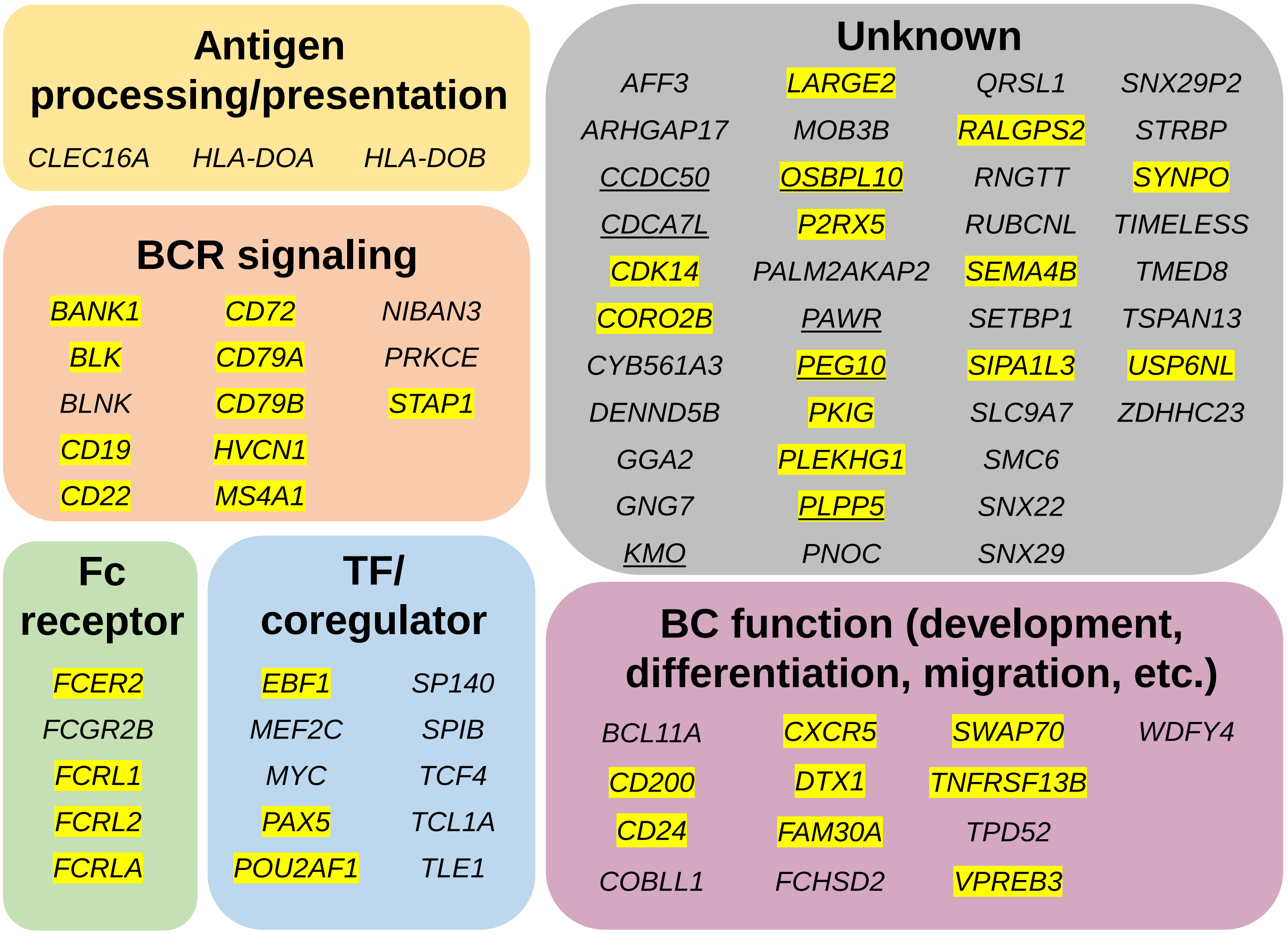

Figure S5: Functional role of B cell signature genes. B cell signature genes (n=85) are categorized based on reported functions in B cells. Genes characterized as having B cell-specific expression based on human ImmGen (https://www.immgen.org/) gene expression data (n=39) are highlighted in yellow. For those with an unknown role in B cells, genes with a reported relationship to B cell-associated cancers are underlined. *, p <0.05; **, p<0.01; ***, p<0.001; ****, p<0.0001.
